# Wild Edible Plant Nutritional Contribution and Consumer Perception in Ethiopia

**DOI:** 10.1155/2020/2958623

**Published:** 2020-09-04

**Authors:** Haile Tesfaye Duguma

**Affiliations:** Department of Postharvest Management, College of Agriculture and Veterinary Medicine, Jimma University, Ethiopia

## Abstract

The scarcity, high cost, and unreliable supply of healthy food in developing countries have resulted in the search for cheap and alternative sources of healthy and nutritious food. Wild edible plants (WEPs) are one of the alternative sources of healthy and nutritious food, and they are crucially important in supporting the global food basket in all parts of the world in general and in sub-Saharan Africa in particular. These wild edible plants have played a significant role in supplying food and nutritional requirements and increasing the health status of poor communities in many rural parts of the world. In Ethiopia, rural communities use wild edible plants as a means of survival especially during times of drought and famine and during other forms calamities and crises. Wild edible plants have high nutritional content, including proteins, vitamin B2, and vitamin C, which can be used as alternatives to conventional plant-based human diets. The available literature has revealed that some wild edible plants also have medicinal properties. Even though wild edible plants are important for food security, they are usually overlooked and perceived as food for poor families. This review indicates that fruits are the most commonly used WEPs, both for consumption and medicinal value, and most plant parts are eaten directly in raw forms. This review focuses on the commercial exploitation of wild edible plants as a source of dietary supplements and alternative medicines and as a means to generate income; it also focuses on consumer perception toward wild edible plants in Ethiopia. Despite easy accessibility and availability, the consumption of wild edible plants is challenged by numerous factors. This review suggests that nutrition policies have to promote the utilization of wild edible plants as one pillar of food and nutrition security. Bioactive compound contents and antinutritional factor contents of wild and medicinal plants need further investigation.

## 1. Introduction

Food and nutrition security is a major challenge that our world is facing nowadays. Approximately two billion people are estimated to suffer from micronutrient deficiencies that make them more susceptible to disease, and this can be a significant obstacle to economic growth [1]. The issue of food security is severe, especially in largely import-dependent countries of sub-Saharan Africa [2]. However, the continent shows highly biodiverse environments with valuable, wild edible plants (WEPs) which are often neglected [3].

The long history of humans' ability to adapt to natural environments and to interact with nature and social circumstances is profoundly devoted to edible wild plants and animals. From the early hunter-gatherers and across different adaptation stages, plants have assumed great importance in human societies and many people all over the world have depended on many wild species particularly for food and medicines (Ferreira et al., 2016).

Wild edible plants are those plants with edible parts that grow naturally on farm land and on fallow or uncultivated land [4–6]. Different wild edible plants have played a significant role in different geographical regions of the world throughout human history [7].

Wild vegetables contribute to people's food security and health in many rural areas of the world [8, 9]. They may have remarkable nutrient values and can be an important source of vitamins, fibers, minerals, and fatty acids; they may also show important medicinal properties [10]. Wild edible plants have always been an essential and widespread food source for food-insecure families living in poverty in developing countries [11, 12]. They are relevant to household food security and nutrition in some rural areas and are relied on to supplement the staple food, to fill seasonal food shortages, and to serve as emergency food during famine [13–17]. They are also important for many communities in rural villages [18, 19] and even those in urban areas, especially among the poor and marginalized [20–22]. Wild edible plants are of crucial importance in all parts of the world in supporting the global food basket [23]. According to Lulekal et al. [24], about one billion people in the world use wild foods (mostly from plants) on a daily basis. Wild edible plants are nutritionally rich and can especially supplement vitamins and micronutrients [25]. They can also supplement nutritional requirements due to their better nutritional value [26, 27].

Besides nutritional value, income and employment can be obtained from the sale or exchange of their fruits, leaves, juice, and local drinks [5]. Although many wild edible plants are used as a food supplement or as a means of survival during drought and famine, the importance of wild edible plants has been overlooked by the majority of the rural population [5, 6, 16, 28].

Ethiopia is the fifth country in tropical Africa in terms of the diversity of flora [29]. In addition, it is known worldwide as one of the global centres of biodiversity where much of this biodiversity is associated with forest resources [30]. The Ethiopian flora contains approximately 6000 species of higher plants of which about 10% are endemic [29]. Ethiopia is known as the biodiversity hotspot and centre of origin and diversification for a significant number of food plants and their wild relatives (Awas, 1997). The wide range of climatic and edaphic conditions permitted the growth of a variety of wild edible plants [31].

Ethiopia is one of the developing countries which depend on wild edible plants to fulfil nutritional needs in addition to domesticated cultivars, especially in poor rural communities during periods of food scarcity [32, 33].

In most parts of Ethiopia, wild edible plants are a recovering part of the feeding habits of many communities [33]. Lulekal et al. [24] noted that about 413 kinds of WEPs are consumed in Ethiopia. Thus, many rural people of Ethiopia usually feed on wild edible plants for survival during food shortages [18]. The findings of this study conducted in Yayo forest show that indigenous fruits, vegetables, and tubers can make a positive contribution to food and nutrition security, as they are well adapted to local environmental conditions. Increasing the production of these underutilized food plants in farmers' home gardens and promoting their consumption could contribute to a more sustainable approach to prevent protein/energy malnutrition and micronutrient deficiencies [34]. Wild vegetables can constitute important local commodities fetching high prices on local and regional markets and as such contribute to local cash income [35, 36].

Wild plants provide medicines that are affordable and readily available to the vast majority of the rural population in Ethiopia, as is the case in many other developing countries in the world. Research has shown that many of the wild edible plants have been found to be rich sources of one or more of the nutritionally important substances, such as proteins, carbohydrates, vitamins, and minerals. Besides the dietary substances, some of them also contain considerable amounts of a variety of health-promoting compounds, such as phenolic compounds [37–39].

The food and nutritional contribution and the medicinal value of wild edible plants have not been investigated fully in Ethiopia. Therefore, the objective of this review is to explore available information about wild edible plants' nutritional contribution, supplementary role, marketability, and medicinal value, as well as to determine consumer perception toward wild edible plants in Ethiopia.

## 2. Material and Methods

The materials for this review were published documents. The author has included all information on WEPs of Ethiopia from well-known sources such as Scopus, Google Scholar, Web of Science, and Mendeley. Data on Ethiopian WEPs such as their scientific names, nutrition and medicinal values, supplementary role, and consumer perception were gathered and compiled after assessing all available Ethiopian ethnobotanical documents. The literature search also addressed WEPs from other countries in order to point out useful research practices that could be used for future ethnobotanical research on WEPs of Ethiopia. After collecting different scientific sources according to different criteria based on topic and academic field, a final inventory of 105 articles was reviewed thoroughly and provided in the reference list. The data obtained is critically reviewed and arranged systematically with reference to their uses. An Excel spreadsheet was used to analyze data using descriptive statistics to identify the number and percentage of commonly utilized plant parts, mode of consumption, marketability, and medicinal value of WEPs.

## 3. Contribution of Wild Edible Plants to Food and Nutrition Security in Ethiopia

Strategies for ensuring food security often advocate for an intensified agriculture that focuses on the enhanced production of major cereals only [1, 40], but the remaining portion of the agricultural sector is quite blind to dietary diversity, thus disrupting healthy food systems [41]. While there is a consensus that wild foods cannot entirely erase the gap between supply and demand, the researchers underline that the difference between supply and demand would be much wider if wild food was absent from our food system [42].

Underutilized edible wild fruits have become a very important part of human nutrition and cannot be overlooked as far as food security, good health, and income generation are concerned [43, 44]. Proximate analysis of some wild edible plants demonstrates that, in many cases, their nutritional quality is comparable to and may be superior to domesticated varieties [31, 33]. Wild edible plants are important nutrient and vitamin supplements for indigenous people [45, 46]. From the nutritional perspective, wild edible plants are sources of many micronutrients commonly lacking in staple-dependent diets in developing countries, which are important for human health and development [47]. Wild edible plants have high nutritional contents such as protein, vitamin B2, and vitamin C, which are used as alternatives to conventional vegetables in the human diet [18].

Tables 1 and 2 indicate the proximate composition and mineral contents of some wild edible plants, respectively; the nutritional value recorded indicate that wild edible plants contain remarkable amounts of nutrients which can meet the recommended daily allowance. According to many sources, the amounts of vitamins, minerals, and other nutrients in wild food is on the average greater than in cultivated foods [48]. Wild fruits have great potential as sources of vital nutrients especially for growing children who are prone to malnutrition and who are key fruit collectors [18]. WEPs are consumed to supplement the nutrition of staple foods which is the solution to hidden hunger (hunger due lack of a balanced diet) [49]. To improve food and nutrition security, focusing on different dimensions of food sources like wild edible food is very crucial.

### 3.1. Supplementary Role of Wild Edible Plants

Wild edible plants are consumed for supplementing staple foods and filling food gaps (drought and famine). A study conducted by Tebkew et al. [50] at Quara District, northwest Ethiopia, indicated that wild edible plants were consumed to supplement staple foods (about 70%) and fill food gaps (drought and famine, about 35%). Wild edible plants (WEPs) provide staple food for indigenous people, serve as complementary food for nonindigenous people, and offer an alternative source of cash income for poor communities [51]. The selection of these edible plants is based on simplicity of processing, good taste, time of availability, and low labour requirement. The general public consumes most of the WEPs as snacks, supplements, or refreshments. Amente [52] indicated that most of the indigenous people of Gumuz People in Kamash Woreda occasionally consider WEPs as famine foods or foods in conditions of starvation. Among the wild edible plants in the study site, around 73.33% were used as supplementary food, while the rest (26.67%) were used as regular food or as meals.

Wild edible plants are relevant to household food security and nutrition in some rural areas, particularly in dry lands, to supplement the staple food, to fill seasonal food shortages, and as emergency food during famine [13, 15–17]. Assefa and Abebe [32] reported that out of 30 identified wild edible tree and shrub species, 15 (50%) are used to supplement the regular food supply. Generally, the literature indicates that wild edible plants are used to supplement regular food.

Studies conducted by Maundu et al. [53] indicated that wild edible plants mostly serve as supplementary foods in different parts of Africa. In many parts of developing countries like Ethiopia, hundreds of wild edible plants are known to be sporadically consumed by rural communities [54]. In Ethiopia, wild edible plants are used as supplementary, seasonal, or survival food sources in many cultural groups, and hence plays a role in combating food insecurity [24]. Wild edible plants provide irreplaceable nutritional contents and economic values to people who depend on them in different parties of Ethiopia [55]. The role of wild edible plants as a supplement to nutritional requirements and as a coping strategy for food shortages is clearly indicated by different scholars [16, 32, 33].

### 3.2. Wild Edible Plants Used as Traditional Medicine

In many countries, medicinal plants are widely used as dietary supplements, as daily foods, and as functional foods, with the aim of promoting health. Wild plants contain a large spectrum of plant secondary metabolic products such as polyphenols, terpenoids, and polysaccharides, which make them good candidates as nutraceuticals, i.e., functional foods which contain potentially health-promoting ingredients [56].

Globally, about 64% of the total world population are reliant on traditional medicine for their healthcare needs [57]. Traditional medicine is the sum total of all knowledge and practices used in diagnosis, prevention, and elimination of health problems and relying exclusively on practical experience and observation passed from generation to generation verbally and in writing [58]. The healing properties of plants have been developed through time by the trial and error experimentation of primitive men and women who struggled with the health problems they encountered, such as the miseries of pain, sickness, and sustained injuries [59].

It is known that many countries in Africa, Asia, and Latin America use traditional medicine (TM) to meet some of their primary health care needs [60]. More than 20,000 plant species are being used in various human cultures around the world for medicinal purposes [61]. Wild fruits have a curing ability for multiple disorders, such as diabetes, cardiovascular problems, inflammations, and digestive and urinary tract disorders, due to their rich sources of antioxidant and fiber components [62, 63].

About 85% of the population in the underdeveloped world do not have access to modern western style health care services (World Health Report, 2008) and rely on traditional medical systems for their health care [64]. Similar results were reported in different countries: a study conducted in Lebanon showed that wild foods were perceived to cure most diseases of human beings [65].

In Ethiopia, about 80% of the human population and 90% of livestock rely on traditional medicine [66, 67]. There are 6000 species of higher plants in Ethiopia out of which more than 14% is used as traditional medicines [68]. Bekele [66] stated that 1000 identified medicinal plant species are reported in the Ethiopian flora. Out of the total wild edible plants found in Berehet District, North Shewa Zone of Amhara Region (Ethiopia), fourteen (23%) nutraceutical wild plants were used to treat 10 (71%) human and 4 (29%) livestock primary health care problems. *Asparagus africanus*, *Carissa spinarum*, *Cordia africana*, and *Ximenia americana* are the most commonly used nutraceutical plants as well as edible plants reported by local people in the Amhara Region, Ethiopia [69]. From the wild edible plants used as traditional medicine, 7 shrubs (50%), 3 herbs (21%), and 4 trees (29%) were the dominant growth forms of the wild edible plants used as traditional medicine in the area. Out of the total nutraceutical plants, leaves (40%) were the most widely used plant parts in the preparation of traditional remedies to treat human and livestock health problems.

Teklehaymanot and Mirutse [70] reported that 70% of the wild edible plants were also used as medicine. Some of the plant parts that are used as a food source but are also ingested as a remedy are taken from the following plants: *Saba comorensis* (Boj.) *Pichon*, *Moringa stenopetala* (Bak. f.) Cuf., *Ximenia americana* L., and *Grewia bicolor* Juss. Kebebew and Leta [71] reported that from the recorded wild edible plants in the study area at Nech Sar National Park, Ethiopia, 18 species serve the local community both as sources of food and as traditional medicine. These nutraceutical plants are used to treat 14 human ailments or health problems. The fruit of *Cordia africana* is mentioned as a treatment for diarrhea. The leaves of *Solanum nigrum* are used to treat abdominal pain, and the roots of *Carissa spinarum* is a remedy for tapeworm. These three important nutraceutical plants are also mentioned in other studies undertaken in different parts of the country [26, 70, 72]. Among the 16 species (32.7%) of WEPs that were grouped as nutraceuticals, the finding indicated that these WEPs were used as food and remedy [73].  Table 3 indicates the list of wild edible plants and their medicinal roles. This showed that wild edible plants are also used for their medicinal value besides their food value. The review indicated that *Moringa stenopetala*, *Solanum nigrum*, *Tamarindus indica*, *Carissa spinarum*, and *Ximenia americana* are some of WEPs commonly used in different parts of the country as indicated by different scholars [49, 50, 71].

### 3.3. Market Values of Wild Edible Plants

Household economic capability to acquire food in the market is a key for the food access pillar [74]. Income and employment can be obtained from the sale or exchange of wild edible fruits, leaves, juice, and local drinks. Income derived from the sale of wild plant species is of particular importance to the poor household. In addition to their use for household consumption, the identified wild edible trees and shrubs are marketable and provide an opportunity to supplement household incomes in the semiarid lowlands of Southern Ethiopia [32]. Wild edible plants also provide other livelihood options in addition to food value. They provide livelihood options in the form of both income generation and subsistence use from different products such as energy construction, shelter/protection, and fodder. Households generate income by selling products in domestic markets and exporting to neighbouring countries, mainly Sudan [49, 50]. According to Balemie and Kebebew [33], income derived from the sale of wild plants is of particular importance to the poorer households, which must supplement food production with cash in order to meet their basic needs.

Abraham in 2016 reported that some of the wild edible trees contributed as additional income sources [75]. For example, *Engulmanzy* and *M. kummel* fruits are harvested mostly freely from the wild and sold at the weekly local and daily urban markets in fresh conditions. Market assessment of wild edible plant species showed that most of the edible plants are not sold only for food purposes but also for other purposes such as timber, agricultural tools, construction, and fuel wood purposes. Some of the wild edible plant part(s) such as the fruits of *Syzygium guineense*, *Balanites aegyptiaca*, *Boswellia neglecta*, and *Ximenia americana* and the leaves of *Moringa stenopetala* are most commonly sold by women and children and provide the opportunity to supplement household income [71]. A study conducted in Tanzania [5] and Ethiopia [31] also revealed that the sale of wild edible plants supplements low farm returns and contributes additional income to households. The marketability of wild edible plants has revealed that the majority of WEPs (75.7%) in East Shewa were not marketed. It has been found that 24.3% were widely marketed from the wild harvest to the local market [26]. The wild edible plants in the Bule Hora Woreda, Southern Ethiopia, are almost not sold as food in the local market except for a few; that is, only *Syzygium guineense* (Willd.) DC. and *Syzygium guineense* var. (Wild.) DC. have been sold in the past during the shortage of food in the area.

But these wild edible plants do not sell in the market nowadays. However, the wild edible plant called *Tamarindus* is still sold in the local market even though there is no food scarcity in the study area [76]. On the other hand, Alemayehu et al. [69] reported that there were no wild edible plants sold in the market at Berehet District, North Shewa Zone of Amhara Region, Ethiopia.

The variation in the marketability of wild edible plants may be due to differences in geographical location, types of wild edible plants, and culture of the society. The list of the most commonly marketed wild edible plants are indicated in Table 4. This review indicates that some wild edible plants (*Tamarindus indica*, *Ximenia americana*, and *Moringa stenopetala*) are marketed in different parts of the country. Besides their food security value, WEPs are sold in the market to generate income.

### 3.4. Plant Parts Used and Mode of Consumption of Wild Edible Plants

#### 3.4.1. Edible Parts of Wild Edible Plants

Fruits, leaves, and gum were the most widely used wild edible plant parts. The parts of wild edible trees and shrubs that are consumed include fruits, seeds, and leaves. Anbessa [76] reported that fruits were the most widely used edible plant parts (79.31), while tubers and fruits (3.45%), young shoots (6.90%), young shoots and fruits (3.45%), roots (3.45%), and gums (3.45%) were the remaining edible parts. Another study indicates that among different parts of medicinal and wild edible plants, fruits are the plant parts that are most widely used for medicinal purposes as well as for edible food [52]. The findings of Kebebew and Leta [71] also indicated that from 51 wild edible plant species, about 40 were fruits, 7 were leaves, and 2 were roots and flower nectars each, while the remaining 3 were seed, young shoot, and stem bark [77]. The figures indicated that different parts of wild edible plants are used for consumption. Leaves, fruits, roots, and seeds are parts of wild edible plants commonly used.

**Figure 1:**
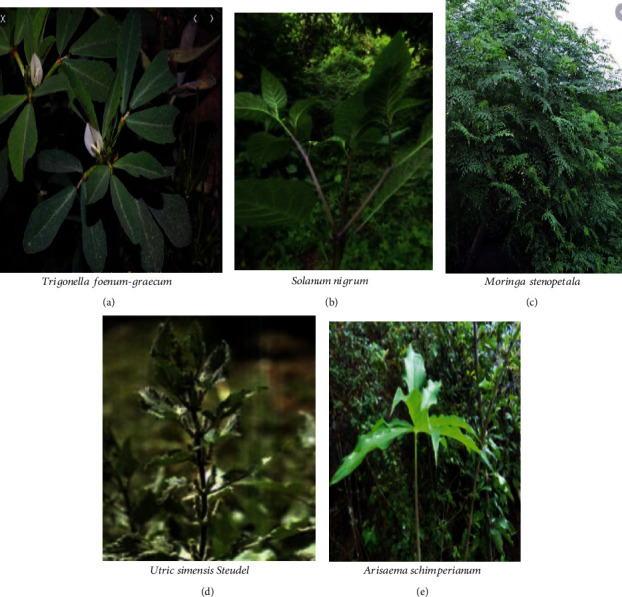
Commonly used wild edible leaves (a–e).

**Figure 2:**
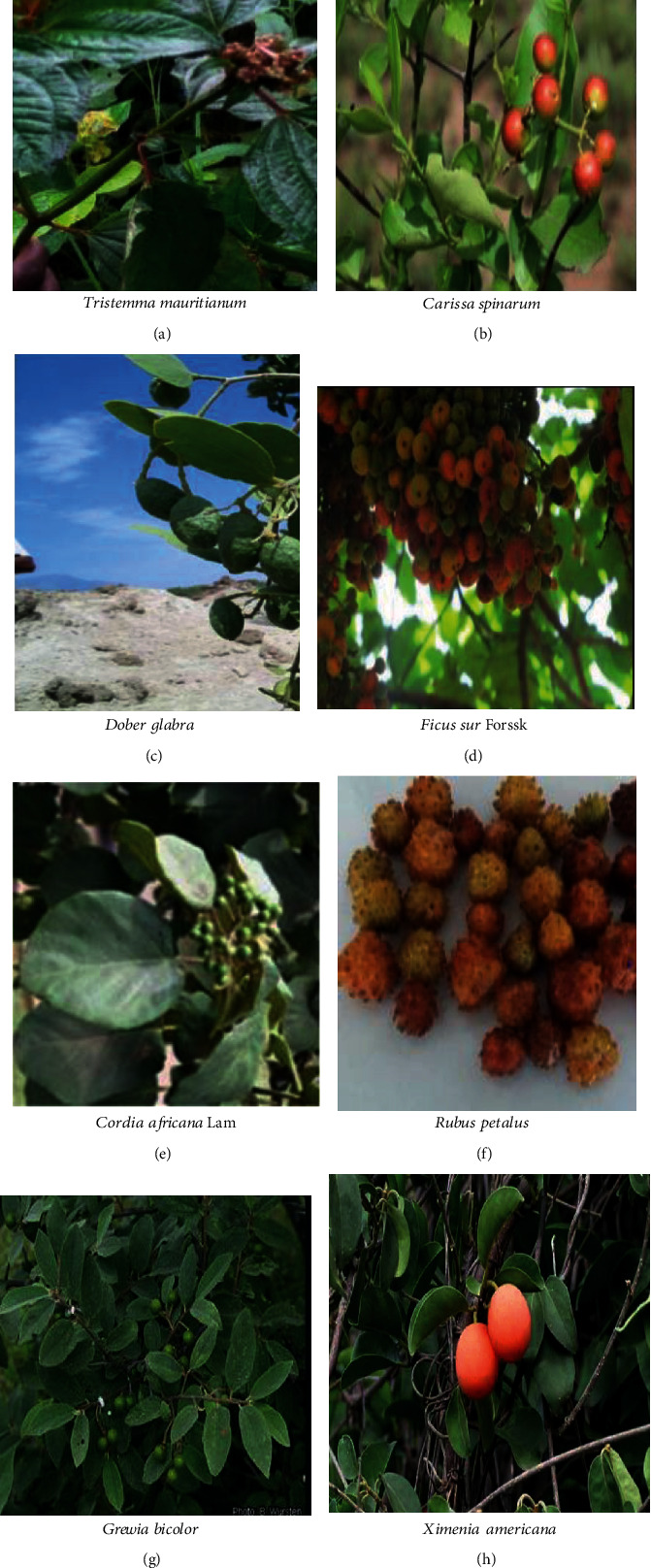
List of commonly consumed wild edible fruits (a–h).

**Figure 3:**
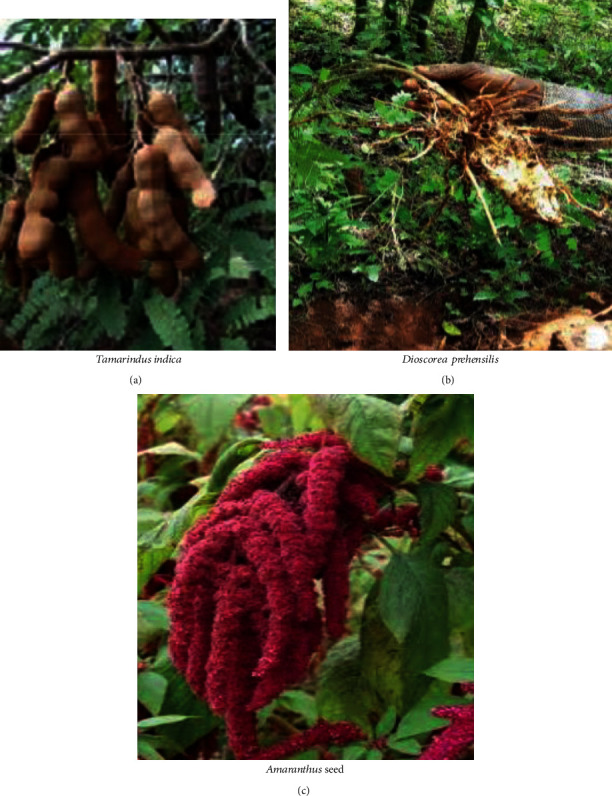
Commonly consumed wild edible tubers (a and b) and seed (c).

Fruit was the dominant part of wild edible plants that was reported to be highly edible in most studies undertaken in different parts of Ethiopia [31, 33, 70, 78]. The increased use of wild fruits compared to other parts of the plant may be because the fruits are used more during seasons of food shortages, and fruit has good taste and flavour because of its chemical composition. However, the result of Amenu [79] and Mesfin et al. [80] indicated that roots are mostly used for medicinal and edible purposes. On the other hand, Guyu and Muluneh [81] reported that 52.4% of the total amount of wild foods came from wild vegetables, which is about 5 times higher than fruits and roots. The variation in consumption of wild edible plant parts might be due to the variation of plant species in adapting to different ecological zones and culture of the people in different areas. Figures 1–3 indicate commonly consumed parts of wild edible plants. Fruits, leaves, tubers, and seeds are parts of wild edible plants consumed in Ethiopia.

#### 3.4.2. Modes of Consumption of Wild Edible Plants

Fruits, leaves, and roots were parts of the plants used for consumption. Most plant parts were eaten directly in fresh forms. The mode of consumption of wild edible plants in Bullen District, Northwest Ethiopia, showed that 57.1% are consumed raw, 16.9% are boiled, 6.5% are consumed in juice form, 9.1% are either consumed raw or boiled, and 5.2% are consumed as porridge/sauce [77]. The study of Beche et al. [82] also indicated that the majority of the recorded edible species were consumed fresh without ripening or processing. The study conducted by Tebkew et al. [50] at Quara District, Northwest Ethiopia, indicated that wild edible plants were consumed fresh, dried and cooked, or prepared in different forms. Majority of the edible plants were consumed fresh, while only nine of them were consumed after drying. In Bule Hora Woreda, Southern Ethiopia, the local people reported that majority of the wild edible plants in the area were consumed raw (about 89.66%), except for *Amaranthus caudatus* L., *Dioscore abulbifera* L., and *Premna schimperi* Engl. whose edible parts can be cooked, boiled, and fermented, respectively, for intake. There was no need for cooking, boiling, or roasting the edible parts for consumption in the majority of wild edible plants in the area [76]. Ashagre et al. [83] also reported that most of the plant parts (87%) were eaten uncooked (raw), while some of them (6.5%) needed processing and cooking to make them suitable for consumption and a few of them (6.5%) could be eaten either cooked or uncooked. Alemayehu et al. [69] reported that about 49 species (92%) of these fruits were reported to be eaten raw, whereas 4 species (8%) were consumed cooked or processed. Different studies indicated that most wild edible plants are consumed raw without further processing (cooking and spicing) in different parts of Ethiopia [71, 76, 77].

Seyoum Aragaw [34] indicated that nearly half of the identified plant species are consumed raw, and many of them are fruits. Others are used after various food preparation techniques. For instance, starchy staples such as tubers have to be cooked to destroy toxic compounds. Leafy vegetables are used after frying and/or boiling or steaming over a fire, and some fruits can be used following non-fire-processing methods like *Rubus apetalus*, which is consumed in the form of a juice. About 52% are used raw as snacks, 19% are cooked, 16% are processed, and 12% of the identified plant species can be used after certain processing and cooking steps. About 1% of the identified plant species can be used raw, cooked, or processed, and hence are of multimodal consumption importance.

### 3.5. Preferences of Wild Edible Plants

The preference of wild edible plants varied; some of the wild edible plants are consumed only during famine and not consumed during normal periods [77]. The individual preference for wild edible plant consumption varies from one locality to another [71]. The condition or time when each plant is consumed also varies. Some plants are consumed always even in the presence of appreciable food stock, while others are consumed only at times of acute food shortage and scarcity ([31]; Assefa and Abebe, 2014; [84]). Plants that are consumed at normal periods are highly valued during periods of food scarcity at all levels. Majority of the wild edible plants in the area were eaten as extra food instead of being served as regular meals. This indicates the use of wild edible plants for other purposes such as materials for house construction and as charcoal during periods when there is sufficient access to cultivated plants.

Likewise, the optional consumption of wild plants was happening in different parts of the world [85]. According to the report of Ashagre et al. [83], wild edible plants are an integral part of the diet of the local people in Burji District, Segan Area Zone of the Southern Nations, Nationalities, and Peoples Region (SNNPR), Ethiopia, at times of both food plenty and scarcity. Most wild edible plants recorded in the Derashe and Kucha Districts of south Ethiopia are consumed both in normal periods and during food scarcity. However, about 27.3% of wild edible plants are consumed only when the preferred alternatives are not available [33].

## 4. Attitudes of Consumers to Wild Edible Plants

There was a strong belief, mainly by the indigenous people, that wild foods have a greater capacity to maintain the good health conditions of those who depend on them [81]. Anbessa [76] indicated that the majority of wild edible plants were eaten as extra food rather than as regular meals. Over 70% of the wild edible plants were consumed during times of food scarcity and starvation, when the supplies of stored food crops were declining progressively [70]. The general public consumes most of the WEPs as snacks, supplements, or refreshments. So, most of the indigenous people of the area occasionally consider the WEPs as famine foods or foods for conditions of starvation. Around 73.33% of the WEPs were used as supplementary food, while the rest (26.67%) were used as regular food or as meals [52]. This implies that most of society consider WEPs as famine food.

Consumption of wild edible plants carries the connotation of belonging to a lower strata in society and is considered an insult, because of lack of knowledge [18]. Similarly, in Kayissa Kebele, South Omo Zone, 10 WEPs are not consumed by the majority of the population except when there is a serious shortage of food affecting all strata of the population from the poorest to the richest [16]. Different studies in Ethiopia show that the local taboos seem to dread being fully depressed to the point of consuming WEPs [18, 49]. Although WEPs play an important role in food security, their utilization was constrained by different factors like cultural ignorance, difficulty of collection, and being choice foods [49]. In a similar study, Addis et al. [86] indicated that despite the fact there was a common understanding and belief that WEPs are important to bridge gaps of food deficiency and serve as food supplements, some individuals in communities consider the use of WEPs as a sign of underdevelopment and poverty. This is a serious threat to conservation and consumption of wild edible plants. On the other hand, Addis et al. [31] stated that wild plants in Ethiopia are used as a source of food both at times of plenty and at times of food shortages. Households have indicated the desire to continue to gathering and hunting for wild foods, but the amount they obtained was very low due because the availability of wild foods have been currently reduced due to substitution with staple crops, wild foods are not culturally acceptable, and the hunting and gathering of wild foods have been legally banned [81]. The literature indicates that the perception of wild edible plants as food varies from location to location, and it also varies with economic status, gender, and age of the consumer.

## 5. Threats and Challenges for Utilization of WEPs

Wild edible plants are threatened with various human and natural factors like land use change (expansion of agricultural lands), developmental activities (road construction and urbanization), habitat destruction (timber harvest, fuel wood collection, and wildfire), drought, overharvesting, and overgrazing. These are among the main factors that reduce the diversity and density of wild edible plants in the study area. The finding of Amente [52] indicated that agricultural activities and drought are the major threatening factors followed by construction, overgrazing, fuel wood collection, and urbanization. Similarly, Kebede et al. [87] reported that deforestation and human encroachment ranked as the 1st and 2nd major factors, respectively, followed by drought and firewood collection in the 3rd and 4th places, respectively. In comparison, Balemie and Kebebew [33] reported that drought takes the major part followed by fuel wood collection and selective cutting for construction.

Despite their accessibility and availability, the utilization of wild edible plants is challenged by numerous factors. Tebkew et al. [50] reported in their findings that the difficulty of collecting, the fast deterioration of products, the perception of being a choice/alternative food, cultural ignorance, and lack of awareness about the nutritional value of the products were the major challenges for the utilization of wild edible plants. Among the challenges, using the products as alternative food and ignorance by culture were the main challenges at about 53% and 44%, respectively. The consumption of WEPs is influenced by different problems: the natural environment where WEPs grow makes them very difficult to collect and their deterioration within a short period reduces the quality and value of WEPs, which might cause people to ignore them and shift to other staple foods [18, 50]. Rapid deterioration of WEPs may be due to improper harvesting and postharvest handling practices.

## 6. Inclusion of Wild Edible Plants in Policy for Nutritional Security

Assimilation of wild edible plants into the diet has much larger implications in terms of environmental sustainability, when the world is plagued with the grave crises of climate change and food insecurity, and could lessen the footprints of agriculture and allow for a shift toward more sustainable food systems [88]. Malnutrition is a major health burden in developing countries, and the recognition that nutritional security and biodiversity are linked is fundamental for enlisting policy support to secure wild food use and preserve habitats for wild edible species [42]. Wild food offers several advantages along this line, i.e., wide diversity, easy access to the local resource base, availability, time-tested reliability, and little or no management [42, 89, 90]. Moreover, the problem of micronutrient deficiency or “hidden hunger” that looms large over the global population cannot be erased by staple crops which lack essential micronutrients [41, 91].

Diets in Ethiopia are low in diversity and are lacking in the critical components of a healthy diet such as fruits and vegetables. The low production of fruits and vegetables and their seasonality and unaffordability constrain Ethiopians from reaping the health benefits provided by regular consumption of fruits and vegetables. Wild edible plants have the potential to greatly improve diets by providing alternative sources of more affordable, nutritionally rich fruits and vegetables, with the added advantage of being available all year round and having the ability to grow in drought-prone, water-stressed areas. However, integrating wild edible fruits and vegetables into markets and designing a functional and efficient value chain is key to ensuring increased availability and consumption [92]. Policies, extension activities, and system sensitivity factors might be wont to identify groups that consume WEPs more frequently, helping NGOs and governments to focus on relevant projects more effectively. This is becoming more important as limited resources restrict food security initiatives in rural areas, despite an increased need for these programs in the face of changing climate and increasingly vulnerable smallholder farms [93]. Moreover, Dejene et al. [94] suggested that due to the diversity of wild edible trees, conservation and management strategies are needed to achieve food security from the use of forest resources. Generally, the review confirmed that food security and agricultural policies have to focus on the contribution of wild edible foods as one pillar to food security.

## 7. Conclusion

The present article is an attempt to review the available information regarding the nutritional contribution, supplementary role, and medicinal value of wild edible plants in Ethiopia and determine consumer attitudes toward them. WEPs have a major contribution to the dietary intake of a community, either for use in times of seasonal food shortage, for filling a food gap and supplementing staple food in normal times, and for use as emergency food during famine. Wild edible plants have the potential to greatly improve food security by providing alternative sources of affordable and nutritious food with the added advantage of being available all year round and being able to grow in water-stressed areas and diverse environmental conditions. Fruits are the dominant parts of wild edible plants used for medicinal purposes as well as for consumption. Besides nutritional contributions, WEPs are used as medicine to treat different human diseases and are sold to generate income for rural people. In spite of its high nutritional contribution, wild edible plants are considered as food for poor families. Wild edible plants demand due consideration to fight food insecurity and improve rural livelihood. Nutrition policies have to promote the utilization of WEPs as part of a strategy to improve food security, nutrition, and livelihoods of rural communities throughout the country. Further investigation is needed on the bioactive compound and antinutritional factor contents of wild edible plants.

## Figures and Tables

**Table 1:** Proximate composition (g/100 g) of some selected wild edible plants.

Scientific name	Moisture (fw)	Ash (dmb)	Ether extractives (dmb)	Protein (dmb)	Crude fiber (dmb)	CH (dmb)	Energy (kcal, dmb)	References
*Dobera glabra* ^b^	28.10	6.79	0.46	16.00	2.41	48.65	253.10	[95]
*Adenia ellenbeckii*	83.6 ± 0.3	13.8 ± 0.1	5.0 ± 0.1	27.7 ± 0.2	10.0 ± 0.6	43.5	174.1	[54]
*Amaranthus graecizans*	72.7 ± 0.3	22.0 ± 0.1	3.9 ± 0.4	28.5 ± 0.2	8.5 ± 0.8	37.1	148.2	
*Balanites aegyptiaca*	63.5 ± 1.2	12.5 ± 0.1	2.5 ± 0.8	28.8 ± 0.4	15.5 ± 0.3	40.7	162.6	
*Celosia argentea*	84.1 ± 0.3	23.9 ± 0.5	2.9 ± 0.8	32.7 ± 0.1	9.8 ± 0.1	30.7	122.9	
*Coccinia grandis*	78.5 ± 0.7	15.2 ± 0.1	3.5 ± 0.1	36.3 ± 0.2	10.1 ± 0.6	34.9	139.6	
*Corchorus trilocularis*	83.9 ± 0.0	15.4 ± 0.8	1.5 ± 0.3	20.4 ± 0.2	11.1 ± 0.9	51.7	206.8	
*Justicia flava*	80.6 ± 1.8	25.6 ± 0.0	2.7 ± 0.4	32.9 ± 0.5	7.5 ± 0.7	31.3	125.4	
*Justicia ladanoides*	73.4 ± 0.5	25.3 ± 0.3	2.9 ± 0.3	25.4 ± 0.4	12.5 ± 0.5	33.9	135.5	
*Launaea intybacea*	80.1 ± 1.3	21.4 ± 0.3	3.7 ± 0.1	24.1 ± 0.2	10.7 ± 0.1	40.1	160.3	
*Leptadenia hastata*	76.9 ± 1.6	13.8 ± 0.2	5.5 ± 0.1	20.3 ± 0.8	14.9 ± 0.2	45.5	182.0	
*Pachycymbium laticoronum*	90.5 ± 0.8	13.2 ± 0.2	3.1 ± 0.5	8.1 ± 0.4	15.1 ± 0.2	60.5	242.1	
*Pentarrhinum insipidum*	77.0 ± 0.8	15.5 ± 0.6	3.3 ± 0.1	32.3 ± 0.2	10.9 ± 0.2	38.0	151.9	
*Portulaca quadrifida*	90.9 ± 0.5	24.6 ± 0.8	3.1 ± 0.2	19.6 ± 0.1	15.9 ± 0.2	36.8	147.3	
*Amorphophallus gomboczianus* ^a^	84.5 ± 0.4	6.0 ± 0.2	0.4 ± 0.1	5.8 ± 0.1	4.3 ± 0.0	83.5	333.8	
*Ximenia caffra* ^b^	61.2 ± 0.6	5.0 ± 0.2	23.6 ± 1.0	21.6 ± 0.1	10.4 ± 1.4	39.4	157.5	
*Coccinia grandis*	87.3 ± 0.5	15.9 ± 0.2	5.2 ± 0.2	28.7 ± 0.4	ND	ND	ND	
*Trigonella foenum-graecum*	89.8 ± 1.1	23.8 ± 0.4	5.8 ± 0.3	28.0 ± 0.7	ND	ND	ND	

All are green vegetables, unless otherwise mentioned; ^a^tuber; ^b^fruit. ND = not determined; CH = carbohydrate.

**Table 2:** Mineral contents (mg/100 g dry weight bases) of selected wild edible plants.

Scientific name	Ca	Cu	Fe	Mg	Mn	Zn	References
*Ziziphus spina-christi*	339.5 + 53.5	1.18 + 0.23	71.99 + 7.85	76.3 + 2.3	ND	2.7 ± 0.4	[96]
*Moringa stenopetala*	792.8 ± 92	3.08 ± 0.8	2.89 + 2.2	ND	ND	0.53 ± 0.8	[97]
*Adenia ellenbeckii*	1239	0.54	16.6	404	7.8	3.1	[54]
*Amaranthus graecizans*	3029	0.65	19.3	2049	7.2	2.3	
*Balanites aegyptiaca*	2487	0.61	13.5	701	3.4	1.2	
*Celosia argentea*	2207	1.39	19.8	824	9.1	2.2	
*Coccinia grandis*	3064	0.60	13.0	433	5.6	2.5	
*Corchorus trilocularis*	1767	0.68	18.6	175	8.4	2.9	
*Justicia flava*	3419	1.48	20.6	547	8.4	2.7	
*Launaea intybacea*	2070	1.45	22.0	437	9.9	3.1	
*Leptadenia hastata*	1699	0.59	14.2	214	4.2	2.0	
*Pachycymbium laticoronum*	1128	0.43	13.2	309	9.8	2.4	
*Pentarrhinum insipidum*	1100	0.41	16.3	183	6.2	2.1	
*Portulaca quadrifida*	2193	0.87	20.1	1094	6.8	2.9	
*Amorphophallus gomboczianus* ^a^	428	0.08	8.72	109	1.9	1.1	
*Ximenia caffra* ^b^	180	0.58	1.9	110	1.1	1.3	
*Trigonella foenum-graecum*	1038	3.2	23.14	203.5	ND	1.0	
*Dobera glabra* ^b^	13.88	ND	ND	1.16	1.66	ND	[95]

All are green vegetables, unless otherwise mentioned; ^a^tuber; ^b^fruit. ND = not determined.

**Table 3:** List of wild edible medicinal plants.

Scientific name	Health benefits	Plant part	References
*Balanites aegyptiaca*	Abdominal pain	Leaf/root	
	Malaria	Root	[77]
	Hypertension	Root	
	Bichawoba		
*Bidens pilosa*	Taneapedis	Leaf	
*Amaranthus hybridus*	Tapeworm	Leaf	
	Tapeworm	Root	
*Carissa spinarum*	Constipation and gonorrhea	Fruit	
	Diarrhea	Fruit	
		Fruit	
*Cordia africana*	Constipation	Fruit	
	Abdominal ache	Fruit	
*Corchorus olitorius*	Diarrhea	Leaf	
*Grewia bicolor*	Venereal disease (syphilis)	Fruit	
	Constipation		
*Gardenia ternifolia*	Abdominal distension	Root	
*Momordica foetida*	Bronchitis	Leaf	
	Ringworm	Sap	
	Diarrhea	Aerial part	
*Portulaca quadrifida*	Abdominal ache, *E. coli*	Aerial part	
*Vernonia amygdalina*	Abdominal pain	Leaf	
*Solanum nigrum*	Abdominal pain	Leaf	
	Malaria	Leaf	
*Tamarindus indica*	Abdominal pain	Fruit	
*Ximenia americana*	Gastritis	Fruit	
	Wound	Fruit	
*Ziziphus abyssinica*	Diarrhea and abdominal pain	Root	
*Aloe camperi*	Idiopathic (likift)	Flower and seed	[98]
*Brassica nigra*	Abortion	Seed	
*Carissa spinarum*	Snake bite	Leaf	
	Rh factor/disease	Leaf	
	Evil eye	Root	
*Ferula communis*	Intestinal pain	Root	
*Ficus palmata*	Impetigo (Kunchir)	Latex	
	Hemorrhoid	Latex	
*Ficus vasta*	Febrile illness	Leaf	
*Gossypium hirsutum*	Malaria	Seed	
*Grewia ferruginea*	Eye injury	Leaf	
*Rubus apetalus*	Evil eye	Root	
*Rumex abyssinicus*	Itching	Root	
*Rumex nepalensis*	Stomach ache	Root	
*Rumex nervosus*	Eye pain	Leaf	
	Ringworm (Agogot)	Stem	
	Human wart	Stem	
*Solanum nigrum*	Gastritis	Leaf	
*Thymus schimperi*	Dry cough	Flower	
	Blood pressure	Leaf	
*Urtica simensis*	Eye injury	Leaf	
*Amaranthus caudata*	Scrofulous sores	Leaf	[71]
*Balanites aegyptiaca*	Abdominal pain and snake bite	Fruit	
*Biden spilosa*	Snake bites and wound	Leaf	
*Cadaba farinosa*	Stomach ache and snake bite	Leaf	
*Capsicum annuum*	Stomach ache	Fruit	
*Citrus aurantifolia*	Stomach ache and hypertension	Fruit	
*Cordia africana*	Diarrhea	Fruit	
*Datura stramonium*	Dandruff	Flower	
*Diospyros abyssinica*	Malaria and dysentery	Leaf	
*Ficus sycomorus*	Hepatitis	Sap	
*Lantana camara*	Mosquito repellent	Leaf	
*Moringa stenopetala*	Hypertension and diabetes	Leaf	
*Solanum nigrum*	Abdominal pain	Leaf	
*Tamarindus indica*	Diarrhea	Fruit	
*Ximenia americana*	Abdominal pain	Fruit	
*Ziziphus mucronata*	Dandruff	Leaf	
*Ziziphus spina-christi*	Dandruff	Leaf	
*Uvaria leptoclada*	Respiratory infection and tuberculosis	Root	[70]
*Saba comorensis*	Venereal disease/syphilis	Fruit	
*Balanite srotundifolia*	Gastrointestinal illness and intestinal parasites	Root	
*Cordia sinensis* Lam	Respiratory infection and tuberculosis	Root	
*Cadaba forinosa* Forssk	Gastrointestinal illness and intestinal parasites	Root	
*Flueggea virosa* (Willd.) *Voigt*	Boils, abscess, and swelling	Root	
*Dobera glabra* (Forssk.) *Poir*	Respiratory infection and tuberculosis	Root	
*Salvadora persica* L.	Respiratory infection and tuberculosis		
*Lycium shawii Roem. & Schult*	Boils, abscesses, and swelling	Root	
*Grewia bicolor*	Venereal disease/syphilis	Fruit	
*Maesa lanceolata* Forssk.	Snake bite	Leaf	[99]
*Rhus glutinosa* Hochst.	Liver disease	Fruit	
*Rhus natalensis Bernh.* ex C. Krauss	Ear ache	Stem, latex	
*Rumex abyssinicus* Jacq.	Hearing failure	Stem, latex	
	Gastritis	Stem, latex	
*Acokanthera schimperi*	Insect allergy	Fruit	
*Balanites aegyptiaca*	Eye disease	Bark	
*Carissa spinarum* L.	Intestinal parasite	Fruit	
*Commelina latifolia*	Toothache	Stem	
*Cordia africana* Lam	Wound	Leaf	
*Datura stramonium* L.	Wound	Root, leaf	
*Moringa stenopetala*	Abdominal pain	Leaf	
	Malaria	Root	
*Rumex nervosus* Vahl.	Ringworm	Latex	
*Senna occidentalis* (L.)	Amoeba	Leaf	
*Tamarindus indica* L.	Malaria	Fruit	
	Evil spirit	Bark	

**Table 4:** List of marketable wild edible plants.

Scientific name of WEPs	Parts marketed	References
*Amaranthus caudata*	Leaf	[71]
*Balanites aegyptiaca*	Fruit	
*Boswellia neglecta*	Fruit	
*Colocasia esculenta*	Root	
*Moringa stenopetala*	Leaf	
*Syzygium guineense*	Fruit	
*Sclerocarya birrea* (A. Rich.) Hochst	Fruit	[32]
*Menya tetraphylla* (Schweinf. Ex Hiern	Fruit	
*Opuntia ficus-indica*	Fruit	
*Adansonia digitata* L.	Fruit	[50]
*Diospyros abyssinica* (Hiem) F. White	Fruit	
*Grewia mollis* Juss.	Fruit	
*Saba comorensi*s (Bo). Pichon	Fruit	
*Tamarindus indica* L.	Fruit	
*Carisa spinarum*	Fruit	[49]
*Corchorusolitorius*	Leaf	
*Diospryos mesiliformis*	Fruit	
*Diospyros abyssinica*	Fruit	
*Ficus sur*	Fruit	
*Hibiscus cannabinus*	Fruit	
*Mimusops kummel*	Seed	
*Saba comorensis*	Fruit	
*Ximenia americana*	Fruit	
*Ziziphus spina-christi*	Fruit	

## Data Availability

The materials described in the manuscript, including all relevant raw data, will be freely available to any scientist wishing to use them for noncommercial purposes.
